# Effect of prenatal genetic counseling on anxiety and test attitudes in pregnant women: a randomized trial

**DOI:** 10.1590/1980-220X-REEUSP-2025-0284en

**Published:** 2026-09-28

**Authors:** Çiler Çokan Dönmez, Tuba Güner Emül

**Affiliations:** 1 Osmaniye Korkut Ata University, Osmaniye, Türkiye.; 2 Mersin University, Faculty of Nursing, Mersin, Türkiye.

**Keywords:** Genetic Counseling, Pregnancy, Anxiety, Attitude, Nursing, Aconselhamento Genético, Gravidez, Ansiedade, Atitude, Enfermagem

## Abstract

**Objective:**

This study aimed to evaluate the effect of prenatal genetic counseling on pregnant women’s anxiety levels and attitudes towards prenatal diagnostic tests.

**Method:**

This randomized controlled trial was conducted on 66 pregnant women who visited the Medical Genetics outpatient clinic of Adana City Hospital between September and December 2022. Data were collected using descriptive information forms, the State-Trait Anxiety Inventory (STAI-S), Prenatal Screening and Diagnostic Tests Attitude Questionnaire (PSDTAQ). Data were analyzed using change-score analysis, linear mixed-effects models including group, time, and group × time interaction, and ANCOVA adjusted for baseline score, income, education, and gestational age. Effect sizes and 95% confidence intervals were also calculated.

**Results:**

After adjustment for baseline values and potential confounders, prenatal genetic counseling significantly reduced STAI-S scores and improved PSDTAQ scores compared with routine care (p < 0.001).

**Conclusion:**

These findings support the potential role of nurse-led prenatal genetic counseling in reducing anxiety and improving attitudes toward prenatal testing. Larger multicenter studies with longer follow-up are required before educational or policy recommendations can be made.

## RESUMO

**Objetivo:**

Avaliar o efeito do aconselhamento genético pré-natal sobre os níveis de ansiedade e as atitudes das gestantes em relação aos testes diagnósticos pré-natais.

**Método:**

Ensaio clínico randomizado realizado com 66 gestantes atendidas no Ambulatório de Genética Médica do Hospital da Cidade de Adana entre setembro e dezembro de 2022. Os dados foram coletados por meio de um formulário de informações descritivas, do Inventário de Ansiedade Estado (STAI-S) e do Questionário de Atitudes em Relação aos Testes de Triagem e Diagnóstico Pré-natal (PSDTAQ). As análises incluíram análise da variação, modelos lineares mistos e ANCOVA ajustada para escore basal, renda, escolaridade e idade gestacional.

**Resultados:**

Após ajuste para os valores basais e potenciais fatores de confusão, o aconselhamento genético pré-natal reduziu significativamente os escores do STAI-S e melhorou significativamente os escores do PSDTAQ em comparação com os cuidados de rotina (p < 0,001).

**Conclusão:**

O aconselhamento genético pré-natal conduzido por enfermeiros mostrou-se eficaz na redução da ansiedade e na melhoria das atitudes das gestantes em relação aos testes diagnósticos pré-natais. Estudos multicêntricos com maior tempo de seguimento são recomendados.

## INTRODUCTION

Prenatal screening and diagnostic tests, implemented during the early stages of pregnancy, provide parents with information regarding genetic diseases or anomalies that may arise in the fetus during the intrauterine period^(1)^. The tests serve to identify concerning biological factors, thereby enabling parents to make informed medical decisions. The aim of prenatal screening and diagnostic tests is to assess the health of the developing fetus or embryo, identify potential risks and genetic disorders as early as possible, and empower expectant parents to make informed decisions regarding pregnancy, childbirth, and postpartum care, allowing them to plan accordingly^(2,3,4)^. According to the 2020 World Health Organization (WHO) report on neonatal congenital anomalies, one in every 33 babies globally is born with such anomalies, resulting in 3.2 million births each year^(5)^. The report highlights an increase in congenital anomaly deaths in Türkiye from 1.23% in 2018 to 1.28% in 2020^(5)^. For the aforementioned reasons, the WHO states prenatal screening and diagnostic tests including chorionic villus biopsy (CVS), amniocentesis, and cordocentesis, are available to enable timely diagnosis, treatment, and care^(5)^. If a fetus is diagnosed with a genetic disease, parents may decide to continue or terminate the pregnancy, and can also benefit from Preimplantation Genetic Diagnosis (PGD) to ensure healthy newborns in future pregnancies. Based on the test results, parents have a legal and conscientious obligation to decide whether to bring their baby into the world or not. In order for parents to make an informed decision about continuing or terminating a pregnancy, they must have the necessary information about the issue at hand^(4,6,7,8)^. Therefore, comprehensive prenatal genetic counseling for the pregnant woman and her partner is crucial^(3,9)^. The American College of Obstetricians and Gynecologists (ACOG) recommends that pregnant women be offered prenatal diagnostic testing and receive detailed prenatal counseling based on the results^(10)^. In a prenatal genetic counseling service, pregnant women must be apprised of diagnostic tests, their purpose, estimated and actual outcomes, interpretation of results, and treatment options. At this stage, the nurse’s primary responsibility is to offer counseling while promoting autonomy and informed decision-making^(8,9,10,11)^. Prenatal diagnostic tests can increase anxiety among pregnant women because they entail intervention, carry uncertainties about the outcomes, and require parental decision-making^(12,13)^. Existing research on prenatal diagnosis and screening tests has shown that prenatal counseling services for pregnant women can reduce anxiety levels and decision-making difficulties, while increasing their knowledge about these tests^(6,14,15,16,17,18)^. Pregnancy is a critical stage in a woman’s life that causes changes in biological, social, and psychological aspects. These changes may lead to increased anxiety during pregnancy. Detection of potential risks during the prenatal period may further exacerbate anxiety levels, culminating in adverse outcomes for the pregnant individual, her partner, the fetus, and the pregnancy process^(19,20,21,22,23)^. Therefore, health professionals should assess potential causes of anxiety during pregnancy and develop and execute interventions to mitigate it^(24,25,26)^.

Prenatal genetic counseling comprises determining the fetus’ genetic anomaly risk status and applicable diagnostic tests and results, as well as alternatives. Prenatal diagnostic tests are offered when there is a high probability of genetic disorders in the fetus. Various genetic disorders that can be diagnosed after birth can also be identified during pregnancy. However, it is crucial to obtain comprehensive prenatal genetic counseling when there is a possibility of congenital anomalies in the fetus. This study aspires to bring attention to this matter by uncovering the impact of prenatal genetic counseling on pregnant women’s anxiety levels and their perceptions of prenatal diagnostic examinations.

Although several descriptive studies have been conducted in Türkiye on the anxiety levels of pregnant women undergoing prenatal diagnostic tests^(12,13)^, there is a limited number of randomized controlled experimental studies on prenatal diagnosis^(19)^. Moreover, no studies have been conducted in the field of nursing in Türkiye, examining the impact of prenatal genetic counseling. The significance of prenatal genetic counseling has increased in today’s world with the help of genetic tests that allow for prenatal diagnosis. The nursing profession must respond to society’s developing and changing needs. Therefore, this randomized controlled trial is the first study in Türkiye to examine how prenatal genetic counseling affects anxiety levels and attitudes of Turkish pregnant women towards prenatal diagnostic tests.

### Hypotheses

The hypotheses of this study are as follows:

**H0:** Prenatal genetic counseling has no significant effect on pregnant women’s anxiety levels or their attitudes toward prenatal diagnostic tests.

**H1a:** Pregnant women who receive prenatal genetic counseling will have significantly lower anxiety levels compared to those who do not receive counseling.

**H1b:** Pregnant women who receive prenatal genetic counseling will have more positive attitudes toward prenatal diagnostic tests compared to those who do not receive counseling.

## METHODS

### Study Design

This study employed a prospective, single-center, randomized, controlled experimental design to investigate the impact of prenatal genetic counseling on anxiety levels and attitudes towards prenatal diagnostic tests among pregnant women. The study was registered with ClinicalTrials.gov (No. NCT05508854).

### Setting and Participants

This randomized controlled trial aimed to investigate the impact of prenatal genetic counseling on anxiety levels and attitudes toward prenatal diagnostic tests among pregnant women. A total of 66 pregnant participants were recruited from the Medical Genetics outpatient clinic of Adana City Hospital between September and December 2022. Participants were randomly assigned, with equal allocation to intervention (n = 33) and control (n = 33) groups.

The inclusion criteria were:

- Pregnant women at 8–11 weeks gestation attending their first antenatal visit,
- Having a singleton pregnancy,
- Planning to continue pregnancy follow-up at Adana City Hospital,
- Able to understand and speak Turkish.

Exclusion criteria included:

- Multiple pregnancies,
- Known psychiatric diagnosis or use of anxiolytic medication,
- Previous prenatal genetic counseling experience,
- Any condition interfering with participation or comprehension.

A flow chart illustrating recruitment, inclusion, randomization, and any exclusions is provided in Figure 1.

**Figure 1:**
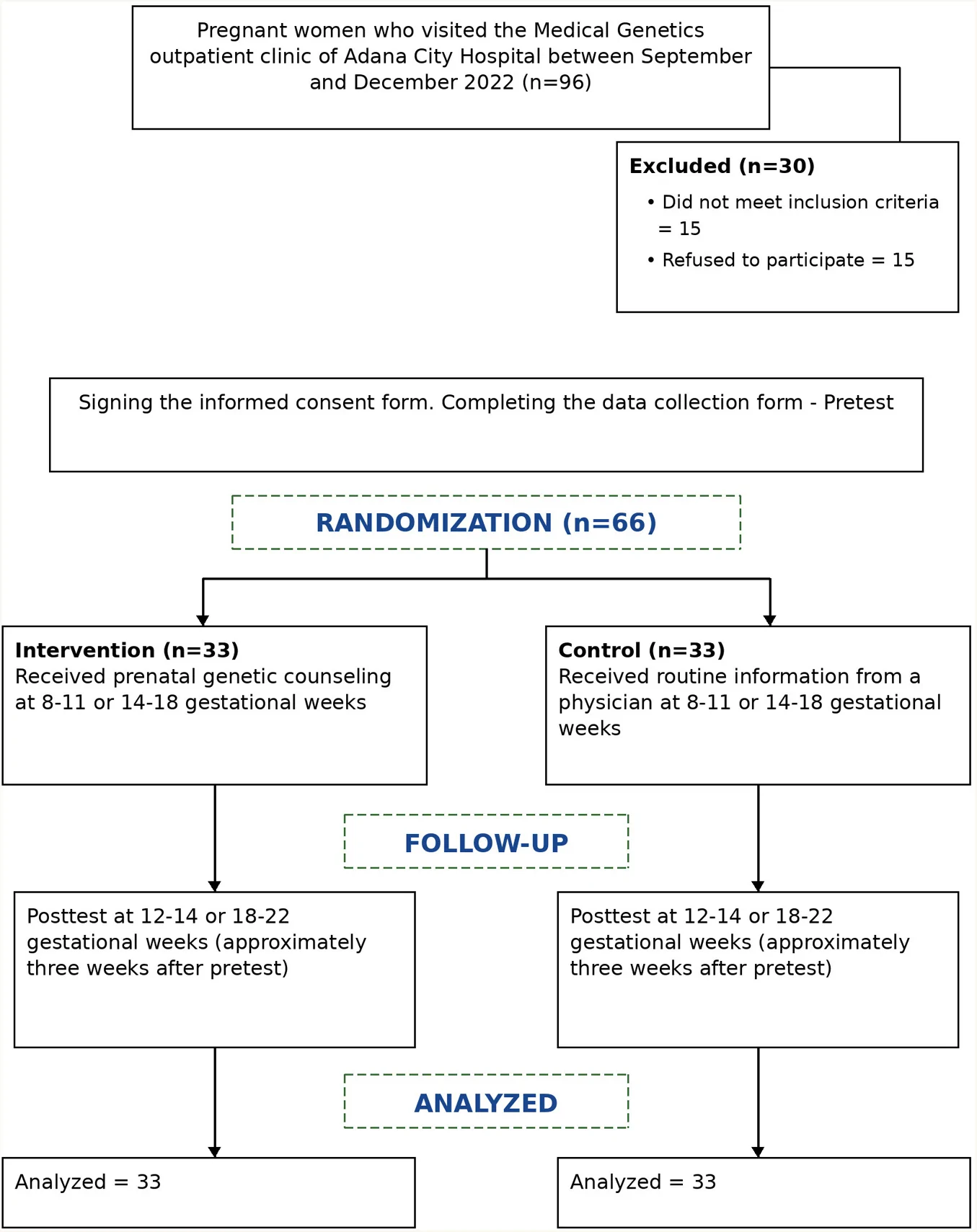
CONSORT flow diagram.

### Sample Size Calculation

Sample size was calculated using G*Power 3.1.9.7 software based on a medium effect size (f = 0.25) according to Cohen’s classification^(20)^, an alpha of 0.05, power of 0.95, and correlation coefficient of 0.50 for repeated measures. The minimum required sample size was 54 (27 per group). Considering potential dropouts, 66 participants were included.

### Data Collection Tools

Descriptive information form, State-Trait Anxiety Inventory (STAI-S) and Prenatal Screening and Diagnostic Tests Attitude Questionnaire (PSDTAQ) were used for data collection.

### Descriptive Information Form

Based on the relevant literature, the form was developed by the researchers to determine the sociodemographic and obstetric characteristics of the pregnant women^(16,17,18)^. It included a total of 18 questions on age, education, income, employment, source of information on prenatal diagnostic test and knowledge of prenatal genetic counseling.

### State-Trait Anxiety Inventory (STAI-S)

The STAI-S was developed by Spielberg et al.^(21)^ and adapted into Turkish by Öner and Le Compte^(22)^. The 20-item inventory measured the level of anxiety on a4-point Likert scale. Each item asks for a rating of agreement on a 4-point Likert scale (1 = not at all; 2 = somewhat; 3 = moderately so; 4 = very much so) for both directly worded items that are indicative of the presence of anxiety (e.g., “I am worried”) and indirectly worded items (e.g., “I am calm”). Possible scores ranged from 20 to 80, with higher scores indicating higher levels of anxiety. The Cronbach’s alpha of this scale ranged between 0.94 – 0.96. The Cronbach’s alpha in our study was 0.77.

### Prenatal Screening and Diagnostic Tests Attitude Questionnaire (PSDTAQ)

The 4-item PSDTAQ was originally developed by Marteau et al.^(23)^ as a measure of informed choice and adapted into Turkish by Yeşilçınar^(19)^ to measure pregnant women’s attitudes toward prenatal screening and diagnostic testing. They were asked to rate the scale statements on a scale of 1 to 7 points, with 1 point indicating a negative attitude and 7 points indicating a very positive attitude. The highest score to be obtained from this scale is 28 and the lowest score is 4, with higher scores indicating positive attitudes. The Cronbach’s alpha of this scale is 0.96. The Cronbach’s alpha of our study was 0.90.

### Interventions

The principal investigator, a specialist nurse with eight years of experience in prenatal genetic counseling at Adana City Hospital Medical Genetics Department, conducted all counseling sessions and data collection. Her expertise includes providing individualized prenatal genetic counseling. This experience ensured consistency and quality in intervention delivery. An educational brochure and PowerPoint presentation were developed after a literature review. The materials covered genetic basics, prenatal diagnostic testing, inheritance patterns, and risk information. Content validity was confirmed by three independent experts in genetics and obstetrics, who were not part of the study team. The pilot test with five pregnant women assessed comprehensibility and acceptability; minor adjustments were made based on their feedback to enhance clarity. In the intervention group, pre-test data (descriptive form, STAI-S, PSDTAQ) were collected before an approximately 30-minute face-to-face counseling session, followed by distribution of the brochure. Posttest assessments were performed at the follow-up visit immediately before disclosure of prenatal screening test results. The control group received standard clinical information from their physicians without additional counseling, with pre- and post-test data collected at similar time points.

Participants in the control group received the standard prenatal care routinely provided in the Medical Genetics outpatient clinic. This consisted of approximately 10–15 minutes of verbal information provided by the attending physician regarding the indication for prenatal screening or diagnostic testing, the procedure itself, and the timing of test results, according to routine clinical practice. No structured counseling protocol, educational brochure, PowerPoint presentation, or additional educational materials were provided to the control group. Therefore, participants in the control group received only the usual care routinely delivered in the clinic without any supplementary nurse-led educational intervention.

### Randomization and Blinding

Participants were randomly allocated to either the intervention or control group using a simple randomization procedure with a 1:1 allocation ratio. The random allocation sequence was generated before participant recruitment by an independent statistician using the Research Randomizer software (https://www.randomizer.org). Allocation concealment was ensured through the use of sequentially numbered, sealed, opaque envelopes, which were prepared by the statistician and opened only after baseline assessment and participant enrollment.

Eligible participants were identified and enrolled by the principal investigator after written informed consent had been obtained and baseline questionnaires had been completed. Following enrollment, the investigator opened the next sealed envelope in sequence to determine group assignment. Because of the nature of the counseling intervention, blinding of participants and the intervention provider was not feasible. However, allocation concealment before assignment minimized selection bias and preserved the integrity of the randomization process.

### Statistical Analysis

SPSS version 24.0 was used for data analysis. Descriptive statistics included number, percentage, mean, standard deviation, minimum and maximum values, median and 25–75% quartiles. Continuous variables were summarized as mean ± SD. Baseline comparisons were performed using independent t-tests, chi-square tests, or Mann–Whitney U tests as appropriate. Because PSDTAQ baseline scores differed significantly between groups, intervention effects were evaluated using change-score analyses and linear mixed-effects models including group, time, and group × time interaction. Linear mixed-effects models were fitted using restricted maximum likelihood estimation, and ANCOVA was performed using the General Linear Model procedure in SPSS. Sensitivity analyses were additionally performed using ANCOVA with posttest scores as dependent variables and baseline score, income, education, and gestational age as covariates. Effect sizes (Cohen’s d and partial η^2^) together with 95% confidence intervals were calculated. Statistical significance was accepted as p < 0.05.

### Ethical Considerations

The study was approved by the clinical research ethics committee of Mersin University (No 485 dated 20/07/2022), Adana City Hospital (No. E-95134008-929, dated 27/07/2022) and Adana Provincial Directorate of Health (No. E-96172664-050.06.04 dated 29/07/2022). All participants were informed about the aim of the study and written informed consent was obtained.

## RESULTS

The mean ages of the pregnant women in the intervention and control groups were 30.0 ± 5.4 and 30.7 ± 6.5, respectively. No statistically significant difference was observed between the two groups in terms of age, education level or employment status (p > 0.05). However, there was a significant difference between the income levels of the intervention and control groups (χ^2^ = 7.142; p = 0.028) (Table 1).

**Table 1:** Socioeconomic characteristics – Adana, Türkiye, 2023.

Variables	Intervention (n = 33) $\bar{\text{X}}$ ± SS			Control (n = 33) $\bar{\text{X}}$ ± SS		Statistical value (p)
Age (years)	30.0 ± 5.4			30.7 ± 6.5		t = -0.476^a^ p = 0.635
	**n**	**%**		**n**	**%**	
**Education**						
Primary school	5	15.2		4	12.1	
Secondary school	7	21.2		5	15.2	χ^2^ = 0.836^b^
High school	10	30.3		13	39.4	p = 0.841
University and above	11	33.3		11	33.3	
**Currently employed**						
Yes	8	24.2		10	30.3	χ^2^ = 0.306^b^
No	25	75.8		23	69.7	p = 0.580
**Income level**						
Lower than expenses	25	75.8		16	48.5	χ^2^ = 7.142^b^
Equal to expenses	7	21.2		17	51.5	p = 0.028
Higher than expenses	1	3.0		–	–	

a: Independent Sample-t test;

b: Chi-squared test.

Regarding obstetric characteristics, 70.8% of the participants in the intervention group had not had a prenatal diagnostic test before, 61.5% had received information about the test, and 78.8% had not received prenatal genetic counseling. The percentages of these three variables for the control group were 60.7%, 62.4% and 75.8%, respectively. There was no statistically significant difference between the intervention and control groups in terms of their experience and sources of information on prenatal diagnostic test and knowledge of prenatal genetic counseling (p > 0.05).

Baseline STAI-S scores did not differ significantly between the intervention and control groups (58.88 ± 7.72 vs. 60.03 ± 5.56, mean difference = −1.15, 95% CI = −4.47 to 2.16, p = 0.490). The mean change from baseline was significantly greater in the intervention group (−25.64 ± 10.14) than in the control group (−6.61 ± 4.33), yielding a between-group change difference of −19.03 points (95% CI = −22.90 to −15.16, p < 0.001; Cohen’s d = −2.44). Consistent with the change-score analysis, posttest STAI-S scores were significantly lower in the intervention group than in the control group (33.24 ± 8.03 vs. 53.42 ± 5.53), corresponding to a between-group difference of −20.18 points (95% CI = −23.58 to −16.78, p < 0.001; Cohen’s d = −2.93) (Table 2).

**Table 2:** Pretest-posttest change scores and effect sizes – Adana, Türkiye, 2023.

Outcome	Time/Change	Intervention (n = 33) Mean ± SD	Control (n = 33) Mean ± SD	Between-group mean difference (95% CI)	p	Cohen’s d
STAI-S	Pretest	58.88 ± 7.72	60.03 ± 5.56	-1.15 (-4.47 to 2.16)	0.490	-0.17
STAI-S	Posttest	33.24 ± 8.03	53.42 ± 5.53	-20.18 (-23.58 to -16.78)	< 0.001	-2.93
STAI-S	Change (post-pre)	-25.64 ± 10.14	-6.61 ± 4.33	-19.03 (-22.90 to -15.16)	< 0.001	-2.44
PSDTAQ	Pretest	16.67 ± 4.75	21.27 ± 2.71	-4.61 (-6.52 to -2.70)	< 0.001	-1.19
PSDTAQ	Posttest	25.45 ± 1.73	23.30 ± 2.11	2.15 (1.20 to 3.10)	< 0.001	1.11
PSDTAQ	Change (post-pre)	8.79 ± 4.23	2.03 ± 2.01	6.76 (5.12 to 8.40)	< 0.001	2.04

Baseline PSDTAQ scores were significantly lower in the intervention group than in the control group (16.67 ± 4.75 vs. 21.27 ± 2.71, mean difference = −4.61, 95% CI = −6.52 to −2.70, p < 0.001). The increase from baseline was significantly greater in the intervention group (8.79 ± 4.23) than in the control group (2.03 ± 2.01), with a between-group change difference of 6.76 points (95% CI = 5.12 to 8.40, p < 0.001; Cohen’s d = 2.04). Consistent with the change-score analysis, posttest PSDTAQ scores were significantly higher in the intervention group than in the control group (25.45 ± 1.73 vs. 23.30 ± 2.11), corresponding to a between-group difference of 2.15 points (95% CI = 1.20 to 3.10, p < 0.001; Cohen’s d = 1.11) (Table 2).

The linear mixed-effects model demonstrated no significant overall group effect (Estimate = −1.05, 95% CI = −4.34 to 2.23, p = 0.529), whereas significant effects of time (Estimate = −6.61, 95% CI = −9.23 to −3.99, p < 0.001) and the group × time interaction (Estimate = −19.03, 95% CI = −22.73 to −15.33, p < 0.001) were observed. The observed group × time interaction showed a large effect size (partial η^2^ = 0.606), indicating that anxiety decreased significantly more over time in the intervention group than in the control group (Table 3).

**Table 3:** Linear mixed-effects model results – Adana, Türkiye, 2023.

Outcome	Effect	Estimate (95% CI)	p value	Partial η^2^
STAI-S	Group	-1.05 (-4.34 to 2.23)	0.529	
STAI-S	Time	-6.61 (-9.23 to -3.99)	< 0.001	
STAI-S	Group × Time	-19.03 (-22.73 to -15.33)	< 0.001	0.606
PSDTAQ	Group	-4.71 (-6.16 to -3.26)	< 0.001	
PSDTAQ	Time	2.03 (0.92 to 3.14)	< 0.001	
PSDTAQ	Group × Time	6.76 (5.18 to 8.33)	< 0.001	0.518

For PSDTAQ, the linear mixed-effects model demonstrated significant effects of group (Estimate = −4.71, 95% CI = −6.16 to −3.26, p < 0.001), time (Estimate = 2.03, 95% CI = 0.92 to 3.14, p < 0.001), and the group × time interaction (Estimate = 6.76, 95% CI = 5.18 to 8.33, p < 0.001). The observed group × time interaction showed a large effect size (partial η^2^ = 0.518), indicating that attitudes toward prenatal screening and diagnostic tests improved significantly more in the intervention group than in the control group (Table 3).

Sensitivity analyses using ANCOVA further confirmed these findings. After adjustment for baseline STAI-S score, income level, education level, and gestational age, the intervention remained significantly associated with lower posttest STAI-S scores (adjusted group effect = −19.97, 95% CI = −23.23 to −16.71, F = 149.84, p < 0.001, partial η^2^ = 0.714) (Table 4).

**Table 4:** ANCOVA sensitivity analyses – Adana, Türkiye, 2023.

Outcome	Adjusted group effect	95% CI	F	p value	Partial η^2^	Covariates
STAI-S posttest	-19.97	-23.23 to -16.71	149.84	< 0.001	0.714	Baseline STAI-S, income, education, gestational age
PSDTAQ posttest	3.15	2.15 to 4.14	40.23	< 0.001	0.401	Baseline PSDTAQ, income, education, gestational age

Similarly, ANCOVA demonstrated that the intervention remained significantly associated with higher posttest PSDTAQ scores after adjustment for baseline PSDTAQ score, income level, education level, and gestational age (adjusted group effect = 3.15, 95% CI = 2.15 to 4.14, F = 40.23, p < 0.001, partial η^2^ = 0.401) (Table 4).

## DISCUSSION

The present study demonstrated a significantly greater reduction in anxiety among women receiving prenatal genetic counseling than among controls. Importantly, this finding remained robust across change-score analyses, linear mixed-effects models, and ANCOVA adjusted for baseline anxiety, income, education, and gestational age. In addition to these findings, the present study represents the first randomized controlled trial conducted in Türkiye to evaluate the effectiveness of nurse led prenatal genetic counseling on anxiety and attitudes toward prenatal screening and diagnostic tests. Previous research in Türkiye has shown that physicians and the internet are the primary sources of information for pregnant women, while nurses play only a limited role in providing prenatal genetic counseling^(19)^. In the present study, only 4% and 3% of participants in the intervention and control groups, respectively, reported nurses as a source of information regarding prenatal diagnostic tests, further highlighting the limited involvement of nurses in prenatal genetic counseling. In contrast, studies from Canada^(27)^, the Netherlands^(28)^, the United States^(6)^, and Switzerland^(29)^ have demonstrated that nurses and midwives actively participate in prenatal genetic counseling. Given the increasing demand for prenatal genetic services and the substantial workload of physicians, expanding the role of nurses in prenatal genetic counseling may improve access to evidence-based counseling and provide more individualized support for pregnant women during decision-making regarding prenatal screening and diagnostic tests. Therefore, the findings of this study contribute to the growing evidence supporting the integration of nurse-led prenatal genetic counseling into routine prenatal care.

The present study demonstrated a significantly greater reduction in anxiety among women receiving prenatal genetic counseling than among controls. Importantly, this finding remained robust across change-score analyses, linear mixed-effects models, and ANCOVA adjusted for baseline anxiety, income, education, and gestational age. The absence of a significant baseline difference in STAI-S scores, together with the significant group × time interaction observed in the mixed-effects model, indicates that the reduction in anxiety was attributable to the counseling intervention rather than to the passage of time or baseline differences. These results confirm H1, demonstrating that genetic counseling has a positive effect on the anxiety levels of pregnant women. Furthermore, various studies in the literature provide supporting evidence for our findings^(8,12,14,18)^. Yeşilçınar discovered that the anxiety level of the intervention group, who received prenatal genetic counseling, was significantly lower than that of the control group^(19)^. Bayat et al. reported that cognitive-behavioral training decreased the anxiety of pregnant women with positive screening results for chromosomal disorders^(30)^. Çakar et al.^(25)^ observed that patients who obtained information from doctors or nurses had significantly lower anxiety levels. Cloutier et al.^(31)^ evaluated the effects of group genetic counseling on high-risk prenatal screening populations and found that women who received individual genetic counseling experienced a significant reduction in anxiety as opposed to group sessions. Mojahed et al.^(32)^ analyzed the effect of education on anxiety of pregnant mothers before amniocentesis and found that the anxiety levels of the intervention group that received the education were significantly lower than the control group. Similarly, the randomized controlled trial conducted by Chuenwattana et al. studying the impact of supportive information on anxiety levels in pregnant women awaiting amniocentesis results revealed significantly lower levels of anxiety in the intervention group^(14)^. The Cronbach’s alpha coefficient for the STAI-S was 0.77 in the present study. Although this value is slightly lower than those reported in previous validation studies, it remains within the acceptable range for research purposes and indicates satisfactory internal consistency for assessing state anxiety in this sample. The study revealed that pregnant women experience anxiety due to potential invasive interventions, worries about fetal health and anomalies, and fear of fetal loss. Prenatal genetic counseling may reduce anxiety by providing individualized information, emotional support, and facilitating informed decision-making during the prenatal testing process.

Although baseline PSDTAQ scores differed significantly between the intervention and control groups, both the linear mixed-effects model and ANCOVA demonstrated that the intervention effect remained statistically significant after adjustment for baseline score, income, education, and gestational age. These findings indicate that the observed improvement in attitudes toward prenatal screening and diagnostic tests was robust and was not explained by baseline imbalances between the groups. Confirming H_1_, this study suggests that prenatal genetic counseling has a positive impact on pregnant women’s attitudes towards prenatal screening and diagnostic tests. Consistent with our results, the only randomized controlled trial to use the PSDTAQ in evaluating the effects of prenatal genetic counseling in Türkiye found a significant increase in participants’ PSDTAQ scores after counseling^(19)^. Therefore, prenatal genetic counseling may improve pregnant women’s attitudes toward prenatal screening and diagnostic tests by facilitating informed and supportive counseling during the decision-making process.

## LIMITATIONS

This study has several limitations. First, it was conducted at a single center with a relatively small sample size, limiting generalizability. Second, participants and the intervention provider could not be blinded, introducing the possibility of attention and expectancy bias. Third, although baseline PSDTAQ scores and income level differed between groups, adjusted analyses using ANCOVA and linear mixed models demonstrated consistent findings. Finally, only short-term outcomes were evaluated. Future studies employing attention-matched control interventions are warranted to distinguish the specific effect of prenatal genetic counseling from the effect of increased professional interaction.

## CONCLUSIONS

The findings of this study suggest that prenatal genetic counseling may be offered during the early stages of pregnancy to support pregnant women undergoing prenatal screening and diagnostic testing. Nurse-led prenatal genetic counseling appears to reduce anxiety and improve attitudes toward prenatal screening in the short term. These findings should be confirmed in larger multicenter randomized trials with longer follow-up before broad educational or policy recommendations are made. Nurses should understand the type, purpose, administration, and duration of prenatal diagnostic assessments, as well as the options available after the results are obtained. The incorporation of prenatal genetic counseling into nursing education should be explored in future educational research.

## Data Availability

The entire dataset supporting the results of this study is available upon request to the corresponding author.
