# Supplementary material for: Effect of prenatal genetic counseling on anxiety and test attitudes in pregnant women: a randomized trial
Source: Rev Esc Enferm USP. 2026 Sep 28;60:e20250284. doi: 10.1590/1980-220X-REEUSP-2025-0284en (PMC13638203; doi:10.1590/1980-220X-REEUSP-2025-0284en)
Supplement: Translation

# Translation: Efecto del asesoramiento genético prenatal sobre la ansiedad y las actitudes hacia las pruebas prenatales en mujeres embarazadas: un ensayo clínico aleatorizado

**DOI:** 10.1590/1980-220X-REEUSP-2025-0284es

**Authors:** Çiler Çokan Dönmez, Tuba Güner Emül

**Affiliations:** 1 Osmaniye Korkut Ata University, Osmaniye, Turquía.; 2 Mersin University, Faculty of Nursing, Mersin, Turquía.

**Keywords:** Asesoramiento Genético, Embarazo, Ansiedad, Actitud, Enfermería

## Abstract

**Objetivo:**

Evaluar el efecto del asesoramiento genético prenatal sobre los niveles de ansiedad y las actitudes de las mujeres embarazadas hacia las pruebas diagnósticas prenatales.

**Método:**

Ensayo clínico aleatorizado realizado con 66 mujeres embarazadas atendidas en la consulta externa de Genética Médica del Hospital de la Ciudad de Adana entre septiembre y diciembre de 2022. Los datos se recopilaron mediante un formulario de información descriptiva, el Inventario de Ansiedad Estado (STAI-S) y el Cuestionario de Actitudes hacia las Pruebas de Cribado y Diagnóstico Prenatal (PSDTAQ). Los datos se analizaron mediante análisis de puntuaciones de cambio, modelos lineales mixtos y ANCOVA ajustada por puntuación basal, ingresos, nivel educativo y edad gestacional.

**Resultados:**

Tras el ajuste por los valores basales y los posibles factores de confusión, el asesoramiento genético prenatal redujo significativamente las puntuaciones del STAI-S y mejoró significativamente las puntuaciones del PSDTAQ en comparación con la atención habitual (p < 0,001).

**Conclusión:**

El asesoramiento genético prenatal dirigido por enfermeras parece eficaz para reducir la ansiedad y mejorar las actitudes de las mujeres embarazadas hacia las pruebas diagnósticas prenatales. Se recomiendan estudios multicéntricos con un seguimiento más prolongado.

## INTRODUCCIÓN

Las pruebas de detección y diagnóstico prenatales, que se realizan durante las primeras etapas del embarazo, brindan a los padres información sobre enfermedades genéticas o anomalías que puedan surgir en el feto durante el período intrauterino^(1)^. Las pruebas sirven para identificar factores biológicos preocupantes, lo que permite a los padres tomar decisiones médicas informadas. El objetivo de las pruebas de detección y diagnóstico prenatal es evaluar la salud del feto o embrión en desarrollo, identificar riesgos potenciales y trastornos genéticos lo más temprano posible, y empoderar a los futuros padres para que tomen decisiones informadas con respecto al embarazo, el parto y la atención posparto, permitiéndoles planificarse en consecuencia^(2,3,4)^. Según el informe de 2020 de la Organización Mundial de la Salud (OMS) sobre anomalías congénitas neonatales, uno de cada 33 bebés a nivel mundial nace con este tipo de anomalías, lo que representa 3,2 millones de nacimientos cada año^(5)^. El informe destaca un aumento en las muertes por anomalías congénitas en Turquía, de 1,23 % en 2018 a 1,28 % en 2020^(5)^. Por las razones antes mencionadas, la OMS afirma que existen pruebas de detección y diagnóstico prenatal, como la biopsia de vellosidades coriónicas (BVC), la amniocentesis y la cordocentesis, que permiten un diagnóstico, tratamiento y atención oportunos^(5)^. Si se diagnostica una enfermedad genética en el feto, los padres pueden decidir continuar o interrumpir el embarazo, y también pueden beneficiarse del diagnóstico genético preimplantacional (DGP) para garantizar el nacimiento de bebés sanos en futuros embarazos. Con base en los resultados de las pruebas, los padres tienen la obligación legal y de conciencia de decidir si traen a su bebé al mundo o no. Para que los padres puedan tomar una decisión informada sobre continuar o interrumpir un embarazo, deben contar con la información necesaria sobre el tema en cuestión^(4,6,7,8)^. Por lo tanto, es fundamental brindar asesoramiento genético prenatal integral a la mujer embarazada y a su pareja^(3,9)^. El Colegio Americano de Obstetras y Ginecólogos (ACOG) recomienda que a las mujeres embarazadas se les ofrezcan pruebas de diagnóstico prenatal y que reciban asesoramiento prenatal detallado basado en los resultados^(10)^. En un servicio de asesoramiento genético prenatal, se debe informar a las mujeres embarazadas sobre las pruebas de diagnóstico, su propósito, los resultados estimados y reales, la interpretación de los resultados y las opciones de tratamiento. En esta etapa, la responsabilidad principal de la enfermera es ofrecer asesoramiento al tiempo que promueve la autonomía y la toma de decisiones informadas^(8,9,10,11)^. Las pruebas de diagnóstico prenatal pueden aumentar la ansiedad entre las mujeres embarazadas porque implican una intervención, conllevan incertidumbres sobre los resultados y requieren que los padres tomen una decisión^(12,13)^. Las investigaciones existentes sobre el diagnóstico prenatal y las pruebas de detección han demostrado que los servicios de asesoramiento prenatal para mujeres embarazadas pueden reducir los niveles de ansiedad y las dificultades para tomar decisiones, al tiempo que aumentan su conocimiento sobre estas pruebas^(6,14,15,16,17,18)^. El embarazo es una etapa crítica en la vida de una mujer que provoca cambios en los aspectos biológicos, sociales y psicológicos. Estos cambios pueden conducir a un aumento de la ansiedad durante el embarazo. La detección de riesgos potenciales durante el período prenatal puede exacerbar aún más los niveles de ansiedad, lo que puede culminar en consecuencias adversas para la mujer embarazada, su pareja, el feto y el proceso del embarazo^(19,20,21,22,23)^. Por lo tanto, los profesionales de la salud deben evaluar las posibles causas de la ansiedad durante el embarazo y desarrollar y llevar a cabo intervenciones para mitigarla^(24,25,26)^.

El asesoramiento genético prenatal consiste en determinar el riesgo de anomalías genéticas del feto, así como las pruebas de diagnóstico aplicables, sus resultados y las alternativas disponibles. Las pruebas de diagnóstico prenatal se ofrecen cuando existe una alta probabilidad de que el feto padezca trastornos genéticos. Durante el embarazo también se pueden identificar diversos trastornos genéticos que, de otro modo, solo se diagnosticarían después del nacimiento. Sin embargo, es fundamental recibir un asesoramiento genético prenatal integral cuando existe la posibilidad de que el feto presente anomalías congénitas. Este estudio aspira a llamar la atención sobre este tema al revelar el impacto del asesoramiento genético prenatal en los niveles de ansiedad de las mujeres embarazadas y en sus percepciones sobre los exámenes de diagnóstico prenatal.

Aunque en Turquía se han realizado varios estudios descriptivos sobre los niveles de ansiedad de las mujeres embarazadas que se someten a pruebas de diagnóstico prenatal^(12,13)^, el número de estudios experimentales aleatorizados y controlados sobre el diagnóstico prenatal es limitado^(19)^. Además, en Turquía no se han realizado estudios en el ámbito de la enfermería que examinen el impacto del asesoramiento genético prenatal. La importancia del asesoramiento genético prenatal ha aumentado en el mundo actual gracias a las pruebas genéticas que permiten el diagnóstico prenatal. La profesión de enfermería debe responder a las necesidades cambiantes y en constante evolución de la sociedad. Por lo tanto, este ensayo controlado aleatorio es el primer estudio realizado en Turquía que examina cómo el asesoramiento genético prenatal afecta los niveles de ansiedad y las actitudes de las mujeres embarazadas turcas hacia las pruebas de diagnóstico prenatal.

### Hipótesis

Las hipótesis de este estudio son las siguientes:

**H0:** El asesoramiento genético prenatal no tiene un efecto significativo en los niveles de ansiedad de las mujeres embarazadas ni en sus actitudes hacia las pruebas de diagnóstico prenatal.

**H1a:** Las mujeres embarazadas que reciben asesoramiento genético prenatal tendrán niveles de ansiedad significativamente más bajos en comparación con aquellas que no reciben asesoramiento.

**H1b:** Las mujeres embarazadas que reciben asesoramiento genético prenatal tendrán actitudes más positivas hacia las pruebas de diagnóstico prenatal en comparación con aquellas que no reciben asesoramiento.

## MÉTODOS

### Diseño del Estudio

Este estudio empleó un diseño experimental prospectivo, unicéntrico, aleatorizado y controlado para investigar el impacto del asesoramiento genético prenatal en los niveles de ansiedad y las actitudes hacia las pruebas de diagnóstico prenatal entre las mujeres embarazadas. El estudio se registró en ClinicalTrials.gov (N.º NCT05508854).

### Contexto y Participantes

Este ensayo controlado aleatorio tuvo como objetivo investigar el impacto del asesoramiento genético prenatal en los niveles de ansiedad y las actitudes hacia las pruebas de diagnóstico prenatal entre las mujeres embarazadas. Se reclutó a un total de 66 participantes embarazadas de la clínica ambulatoria de Genética Médica del Hospital de la Ciudad de Adana entre septiembre y diciembre de 2022. Las participantes fueron asignadas al azar, con una distribución equitativa entre el grupo de intervención (n = 33) y el grupo de control (n = 33).

Los criterios de inclusión fueron:

- Mujeres embarazadas entre las semanas 8 y 11 de gestación que asistieran a su primera consulta prenatal,
- Que tuvieran un embarazo único,
- Que tuvieran previsto continuar el seguimiento del embarazo en el Hospital de la Ciudad de Adana,
- Que pudieran entender y hablar turco.

Los criterios de exclusión incluyeron:

- Embarazos múltiples,
- Diagnóstico psiquiátrico conocido o uso de medicamentos ansiolíticos,
- Experiencia previa en asesoramiento genético prenatal,
- Cualquier afección que interfiriera con la participación o la comprensión.

En la Figura 1 se presenta un diagrama de flujo que ilustra el reclutamiento, la inclusión, la aleatorización y las exclusiones.

**Figura 1:** Flujograma CONSORT.

### Cálculo del Tamaño de la Muestra

El tamaño de la muestra se calculó utilizando el software G*Power 3.1.9.7, basándose en un tamaño de efecto medio (f = 0,25) según la clasificación de Cohen^(20)^, un nivel de significación (alfa) de 0,05, una potencia de 0,95 y un coeficiente de correlación de 0,50 para medidas repetidas. El tamaño mínimo de muestra requerido fue de 54 (27 por grupo). Teniendo en cuenta los posibles abandonos, se incluyeron 66 participantes.

### Herramientas de Recolección de Datos

Para la recolección de datos se utilizaron el formulario de información descriptiva, el Inventario de Ansiedad Estado-Rasgo (State-Trait Anxiety Inventory STAI-S) y el Cuestionario de Actitudes hacia las Pruebas de Detección y Diagnóstico Prenatales (Prenatal Screening and Diagnostic Tests Attitude Questionnaire – PSDTAQ).

### Formulario de Información Descriptiva

Basándose en la literatura relevante, los investigadores elaboraron el formulario para determinar las características sociodemográficas y obstétricas de las mujeres embarazadas^(16,17,18)^. Incluía un total de 18 preguntas sobre edad, nivel educativo, ingresos, empleo, fuente de información sobre las pruebas de diagnóstico prenatal y conocimiento del asesoramiento genético prenatal.

### Inventario de Ansiedad Estado-Rasgo (*State-Trait Anxiety Inventory* – STAI-S)

El STAI-S fue desarrollado por Spielberg et al.^(21)^ y adaptado al turco por Öner y Le Compte^(22)^. El inventario de 20 ítems medía el nivel de ansiedad en una escala de Likert de 4 puntos. Cada ítem solicita una calificación de acuerdo en una escala de Likert de 4 puntos (1 = para nada; 2 = algo; 3 = moderadamente; 4 = mucho) tanto para los ítems redactados de manera directa que indican la presencia de ansiedad (p. ej., «Estoy preocupada») como para los redactados de manera indirecta (p. ej., «Estoy tranquila»). Las puntuaciones posibles oscilaban entre 20 y 80, y las puntuaciones más altas indicaban niveles más elevados de ansiedad. El alfa de Cronbach de esta escala osciló entre 0,94 y 0,96.El alfa de Cronbach de nuestro estudio fue de 0,77.

### Cuestionario de Actitudes Hacia las Pruebas de Detección y Diagnóstico Prenatales (*Prenatal Screening and Diagnostic Tests Attitude Questionnaire* – PSDTAQ)

El PSDTAQ, compuesto por 4 ítems, fue desarrollado originalmente por Marteau et al.^(23)^ como una medida de la elección informada y adaptado al turco por Yeşilçınar^(19)^ para medir las actitudes de las mujeres embarazadas hacia las pruebas de detección y diagnóstico prenatales. Se les pidió que calificaran las afirmaciones de la escala en una escala de 1 a 7 puntos, donde 1 punto indicaba una actitud negativa y 7 puntos indicaban una actitud muy positiva. La puntuación máxima que se puede obtener en esta escala es 28 y la mínima es 4; las puntuaciones más altas indican actitudes positivas. El alfa de Cronbach de esta escala es de 0,96. El alfa de Cronbach de nuestro estudio fue de 0,90.

### Intervenciones

La investigadora principal, una enfermera especialista con ocho años de experiencia en asesoramiento genético prenatal en el Departamento de Genética Médica del Hospital de la Ciudad de Adana, llevó a cabo todas las sesiones de asesoramiento y la recolección de datos. Su experiencia incluye la prestación de asesoramiento genético prenatal individualizado. Esta experiencia garantizó la coherencia y la calidad en la ejecución de la intervención. Se elaboraron un folleto educativo y una presentación en PowerPoint tras una revisión de la literatura. Los materiales abarcaban conceptos básicos de genética, pruebas de diagnóstico prenatal, patrones de herencia e información sobre riesgos. La validez del contenido fue confirmada por tres expertos independientes en genética y obstetricia, quienes no formaban parte del equipo de estudio. La prueba piloto con cinco mujeres embarazadas evaluó la comprensibilidad y la aceptabilidad; se realizaron ajustes menores basados en sus comentarios para mejorar la claridad. En el grupo de intervención, se recolectaron los datos pre-test (formulario descriptivo, STAI-S, PSDTAQ) antes de una sesión de asesoramiento presencial de aproximadamente 30 minutos, seguida de la distribución del folleto. Las evaluaciones post-test se realizaron en la visita de seguimiento inmediatamente antes de la divulgación de los resultados de las pruebas de detección prenatal. El grupo de control recibió información clínica estándar de sus médicos sin asesoramiento adicional, y los datos pre-test y post-test se recolectaron en momentos similares.

Las participantes del grupo de control recibieron la atención prenatal estándar que se brinda habitualmente en la clínica ambulatoria de Genética Médica. Esta consistió en aproximadamente 10 a 15 minutos de información verbal proporcionada por el médico de atención sobre la indicación de las pruebas de detección o diagnóstico prenatal, el procedimiento en sí y el momento en que se obtendrían los resultados, de acuerdo con la práctica clínica habitual. No se proporcionó al grupo de control ningún protocolo estructurado de asesoramiento, folleto educativo, presentación de PowerPoint ni materiales educativos adicionales. Por lo tanto, las participantes del grupo de control recibieron únicamente la atención habitual que se brinda de manera rutinaria en la clínica, sin ninguna intervención educativa complementaria a cargo de enfermeras.

### Aleatorización y Cegamiento

Las participantes fueron asignadas al azar al grupo de intervención o al grupo de control mediante un procedimiento de aleatorización simple con una proporción de asignación de 1:1. La secuencia de asignación aleatoria fue generada antes del reclutamiento de las participantes por un estadístico independiente utilizando el software Research Randomizer (https://www.randomizer.org). El ocultamiento de la asignación se garantizó mediante el uso de sobres opacos, sellados y numerados secuencialmente, los cuales fueron preparados por el estadístico y abiertos únicamente después de la evaluación inicial y la inscripción de los participantes.

Los participantes elegibles fueron identificados e inscritos por el investigador principal después de que se obtuviera el consentimiento informado por escrito y se completaran los cuestionarios iniciales. Tras la inscripción, el investigador abrió el siguiente sobre sellado en la secuencia para determinar la asignación a los grupos. Debido a la naturaleza de la intervención de asesoramiento, no fue factible el enmascaramiento de los participantes ni del proveedor de la intervención. Sin embargo, el ocultamiento de la asignación antes de la distribución minimizó el sesgo de selección y preservó la integridad del proceso de aleatorización.

### Análisis Estadístico

Se utilizó la versión 24.0 del SPSS para el análisis de datos. Las estadísticas descriptivas incluyeron número, porcentaje, media, desviación estándar, valores mínimos y máximos, mediana y cuartiles del 25 al 75 %. Las variables continuas se resumieron como media ± DE. Las comparaciones con los valores iniciales se realizaron mediante pruebas t independientes, pruebas de chi-cuadrado o pruebas U de Mann–Whitney, según correspondiera. Dado que las puntuaciones iniciales del PSDTAQ diferían significativamente entre los grupos, los efectos de la intervención se evaluaron mediante análisis de la variación en las puntuaciones y modelos lineales de efectos mixtos que incluían el grupo, el tiempo y la interacción grupo × tiempo. Los modelos lineales de efectos mixtos se ajustaron mediante estimación de máxima verosimilitud restringida, y se realizó un ANCOVA utilizando el procedimiento del Modelo Lineal General en SPSS. Además, se realizaron análisis de sensibilidad utilizando el ANCOVA con las puntuaciones de la Post test como variables dependientes y la puntuación inicial, los ingresos, el nivel educativo y la edad gestacional como covariables. Se calcularon los tamaños del efecto (d de Cohen y η^2^ parcial) junto con los intervalos de confianza del 95 %. Se consideró estadísticamente significativo un valor de p < 0,05.

### Consideraciones Éticas

El estudio fue aprobado por el comité de ética de investigación clínica de la Universidad de Mersin (n.º 485, con fecha del 20/07/2022), elHospital de la Ciudad de Adana (n.º E-95134008-929, del 27/07/2022) y la Dirección Provincial de Salud de Adana (n.º E-96172664-050.06.04, del 29/07/2022). Se informó a todas las participantes sobre el objetivo del estudio y se obtuvo su consentimiento informado por escrito.

## RESULTADOS

Las edades medias de las mujeres embarazadas de los grupos de intervención y de control fueron de 30,0 ± 5,4 y 30,7 ± 6,5, respectivamente. No se observaron diferencias estadísticamente significativas entre ambos grupos en cuanto a edad, nivel educativo o situación laboral (p > 0,05). Sin embargo, sí se observó una diferencia significativa entre los niveles de ingresos de los grupos de intervención y de control (χ^2^ = 7,142; p = 0,028) (Tabla 1).

**Tabla 1:** Características socioeconómicas – Adana, Turquía, 2023.

Variables	Intervención (n = 33) $\bar{\text{X}}$ ± SS		Grupo de control (n = 33) $\bar{\text{X}}$ ± SS		Valor estadístico (p)
Edad (años)	30,0 ± 5,4		30,7 ± 6,5		t = -0,476^a^ p = 0,635
	**n**	**%**	**n**	**%**	
**Educación**					
Educación primaria	5	15,2	4	12,1	
Educación secundaria	7	21,2	5	15,2	χ^2^ = 0,836^b^
Bachillerato	10	30,3	13	39,4	p = 0,841
Universidad y estudios superiores	11	33,3	11	33,3	
**Actualmente en activo**					
Sí	8	24,2	10	30,3	χ^2^ = 0,306^b^
No	25	75,8	23	69,7	p = 0,580
**Nivel de ingresos**					
Inferior a los gastos	25	75,8	16	48,5	χ^2^ = 7,142^b^
Igual a los gastos	7	21,2	17	51,5	p = 0,028
Superior a los gastos	1	3,0	–	–	

a: Prueba t para muestras independientes;

b: Prueba de chi-cuadrado.

En cuanto a las características obstétricas, el 70,8 % de las participantes del grupo de intervención no se había sometido anteriormente a una prueba de diagnóstico prenatal, el 61,5 % había recibido información sobre la prueba y el 78,8 % no había recibido asesoramiento genético prenatal. Los porcentajes de estas tres variables para el grupo de controlfueron 60,7 %, 62,4 % y 75,8 %, respectivamente. No se observaron diferencias estadísticamente significativas entre los grupos de intervención y de controlen cuanto a su experiencia, las fuentes de información sobre las pruebas de diagnóstico prenatal y el conocimiento del asesoramiento genético prenatal (p > 0,05).

Las puntuaciones iniciales del STAI-S no mostraron diferencias significativas entre el grupo de intervención y el grupo de control (58,88 ± 7,72 frente a 60,03 ± 5,56; diferencia media = −1,15; IC del 95 % = −4,47 a 2,16; p = 0,490). El cambio medio con respecto al inicio fue significativamente mayor en el grupo de intervención (−25,64 ± 10,14) que en el grupo de control (−6,61 ± 4,33), lo que supuso una diferencia de cambio entre grupos de −19,03 puntos (IC del 95 % = −22,90 a −15,16, p < 0,001; d de Cohen = −2,44). En consonancia con el análisis de la puntuación de cambio, las puntuaciones del STAI-S tras la prueba fueron significativamente más bajas en el grupo de intervención que en el grupo de control (33,24 ± 8,03 frente a 53,42 ± 5,53), lo que corresponde a una diferencia entre grupos de −20,18 puntos (IC del 95 % = −23,58 a −16,78, p < 0,001; d de Cohen = −2,93) (Tabla 2).

**Tabla 2:** Puntuaciones de cambio entre la pre test y la post test y tamaños del efecto – Adana, Turquía, 2023.

Resultado	Momento/Cambio	Intervención (n = 33) Media ± DE	Grupo de control (n = 33) Media ± DE	Diferencia de medias entre grupos (IC del 95 %)	p	d de Cohen
STAI-S	Pre test	58,88 ± 7,72	60,03 ± 5,56	-1,15 (-4,47 a 2,16)	0,490	-0,17
STAI-S	Post test	33,24 ± 8,03	53,42 ± 5,53	-20,18 (-23,58 a -16,78)	< 0,001	-2,93
STAI-S	Variación (posterior-previo)	-25,64 ± 10,14	-6,61 ± 4,33	-19,03 (-22,90 a -15,16)	< 0,001	-2,44
PSDTAQ	Pre test	16,67 ± 4,75	21,27 ± 2,71	-4,61 (-6,52 a -2,70)	< 0,001	-1,19
PSDTAQ	Post test	25,45 ± 1,73	23,30 ± 2,11	2,15 (1,20 a 3,10)	< 0,001	1,11
PSDTAQ	Variación (posterior-anterior)	8,79 ± 4,23	2,03 ± 2,01	6,76 (5,12 a 8,40)	< 0,001	2,04

Las puntuaciones iniciales del PSDTAQ fueron significativamente más bajas en el grupo de intervención que en el grupo de control (16,67 ± 4,75 frente a 21,27 ± 2,71; diferencia media = −4,61; IC del 95 % = −6,52 a −2,70; p < 0,001). El aumento con respecto al valor inicial fue significativamente mayor en el grupo de intervención (8,79 ± 4,23) que en el grupo de control (2,03 ± 2,01), con una diferencia de cambio entre grupos de 6,76 puntos (IC del 95 % = 5,12 a 8,40, p < 0,001; d de Cohen = 2,04). En consonancia con el análisis de la puntuación de cambio, las puntuaciones del PSDTAQ tras la prueba fueron significativamente más altas en el grupo de intervención que en el grupo de control (25,45 ± 1,73 frente a 23,30 ± 2,11), lo que corresponde a una diferencia entre grupos de 2,15 puntos (IC del 95 % = 1,20 a 3,10, p < 0,001; d de Cohen = 1,11) (Tabla 2).

El modelo lineal de efectos mixtos no mostró ningún efecto global significativo del grupo (estimación = −1,05; IC del 95 % = −4,34 a 2,23; p = 0,529), mientras que sí se observaron efectos significativos del tiempo (estimación = −6,61, IC del 95 % = −9,23 a −3,99, p < 0,001) y de la interacción grupo × tiempo (estimación = −19,03, IC del 95 % = −22,73 a −15,33, p < 0,001). La interacción grupo × tiempo observada mostró un tamaño del efecto elevado (η^2^ parcial = 0,606), lo que indica que la ansiedad disminuyó significativamente más con el tiempo en el grupo de intervención que en el grupo de control (Tabla 3).

**Tabla 3:** Resultados del modelo lineal de efectos mixtos – Adana, Turquía, 2023.

Resultado	Efecto	Estimación (IC del 95 %)	Valor p	η^2^ parcial
STAI-S	Grupo	-1,05 (-4,34 a 2,23)	0,529	
STAI-S	Tiempo	-6,61 (-9,23 a -3,99)	< 0,001	
STAI-S	Grupo × Tiempo	-19,03 (-22,73 a -15,33)	< 0,001	0,606
PSDTAQ	Grupo	-4,71 (-6,16 a -3,26)	< 0,001	
PSDTAQ	Tiempo	2,03 (0,92 a 3,14)	< 0,001	
PSDTAQ	Grupo × Tiempo	6,76 (5,18 a 8,33)	< 0,001	0,518

En cuanto al PSDTAQ, el modelo lineal de efectos mixtos demostró efectos significativos del grupo (estimación = −4,71; IC del 95 % = −6,16 a −3,26, p < 0,001), del tiempo (estimación = 2,03; IC del 95 % = 0,92 a 3,14; p < 0,001) y de la interacción grupo × tiempo (estimación = 6,76; IC del 95 % = 5,18 a 8,33; p < 0,001). La interacción observada entre grupo y tiempo mostró un tamaño del efecto elevado (η^2^ parcial = 0,518), lo que indica que las actitudes hacia las pruebas de cribado y diagnóstico prenatales mejoraron significativamente más en el grupo de intervención que en el grupo de control (Tabla 3).

Los análisis de sensibilidad realizados mediante ANCOVA confirmaron aún más estos hallazgos. Tras ajustar por la puntuación inicial del STAI-S, el nivel de ingresos, el nivel educativo y la edad gestacional, la intervención siguió estando significativamente asociada con puntuaciones más bajas del STAI-S en la Post test (efecto ajustado del grupo = −19,97; IC del 95 % = −23,23 a −16,71; F = 149,84; p < 0,001; η^2^ parcial = 0,714) (Tabla 4).

**Tabla 4:** Análisis de sensibilidad de ANCOVA – Adana, Turquía, 2023.

Resultado	Efecto ajustado por grupo	IC del 95 %	F	Valor p	η^2^ parcial	Covariables
Post test del STAI-S	-19,97	-23,23 a -16,71	149,84	< 0,001	0,714	Puntuación inicial del STAI-S, ingresos, nivel educativo, edad gestacional
Post test del PSDTAQ	3,15	2,15 a 4,14	40,23	< 0,001	0,401	PSDTAQ inicial, ingresos, nivel educativo, edad gestacional

De manera similar, el ANCOVA demostró que la intervención se mantuvo significativamente asociada con puntuaciones más altas en el PSDTAQ tras la prueba, después de ajustar por la puntuación inicial del PSDTAQ, el nivel de ingresos, el nivel educativo y la edad gestacional (efecto de grupo ajustado = 3,15; IC del 95 % = 2,15 a 4,14; F = 40,23; p < 0,001; η^2^ parcial = 0,401) (Tabla 4).

## DISCUSIÓN

El presente estudio demostró una reducción significativamente mayor de la ansiedad entre las mujeres que recibieron asesoramiento genético prenatal en comparación con el grupo de control. Es importante destacar que este hallazgo se mantuvo sólido en los análisis de puntuaciones de cambio, los modelos lineales de efectos mixtos y el ANCOVA ajustado por la ansiedad inicial, los ingresos, el nivel educativo y la edad gestacional. Además de estos hallazgos, el presente estudio constituye el primer ensayo controlado aleatorio realizado en Turquía para evaluar la eficacia del asesoramiento genético prenatal a cargo de enfermeras en la ansiedad y las actitudes hacia las pruebas de detección y diagnóstico prenatales. Investigaciones previas en Turquía han demostrado que los médicos e Internet son las principales fuentes de información para las mujeres embarazadas, mientras que las enfermeras desempeñan solo un papel limitado en la prestación de asesoramiento genético prenatal^(19)^. En el presente estudio, solo el 4 % y el 3 % de las participantes en los grupos de intervención y control, respectivamente, mencionaron a las enfermeras como fuente de información sobre las pruebas de diagnóstico prenatal, lo que resalta aún más la limitada participación de las enfermeras en el asesoramiento genético prenatal. Por el contrario, estudios de Canadá^(27)^, los Países Bajos^(28)^, Estados Unidos^(6)^ y Suiza^(29)^ han demostrado que las enfermeras y las parteras participan activamente en el asesoramiento genético prenatal. Dada la creciente demanda de servicios genéticos prenatales y la considerable carga de trabajo de los médicos, ampliar el papel de las enfermeras en el asesoramiento genético prenatal podría mejorar el acceso a un asesoramiento basado en la evidencia y brindar un apoyo más individualizado a las mujeres embarazadas durante la toma de decisiones relacionadas con las pruebas de detección y diagnóstico prenatales. Por lo tanto, los hallazgos de este estudio contribuyen a la creciente evidencia que respalda la integración del asesoramiento genético prenatal a cargo de enfermeras en la atención prenatal de rutina.

Los resultados demostraron una reducción significativamente mayor de la ansiedad entre las mujeres que recibieron asesoramiento genético prenatal en comparación con el grupo de control. Es importante destacar que este hallazgo se mantuvo sólido en los análisis de puntuaciones de cambio, los modelos lineales de efectos mixtos y el ANCOVA ajustado por la ansiedad inicial, los ingresos, el nivel educativo y la edad gestacional. La ausencia de una diferencia significativa en las puntuaciones del STAI-S al inicio del estudio, junto con la interacción significativa entre grupo y tiempo observada en el modelo de efectos mixtos, indica que la reducción de la ansiedad fue atribuible a la intervención de asesoramiento y no al paso del tiempo ni a diferencias iniciales. Estos resultados confirman la hipótesis H1, demostrando que el asesoramiento genético tiene un efecto positivo en los niveles de ansiedad de las mujeres embarazadas. Además, diversos estudios en la literatura proporcionan evidencia que respalda nuestros hallazgos^(8,12,14,18)^. Yeşilçınar descubrió que el nivel de ansiedad del grupo de intervención, que recibió asesoramiento genético prenatal, fue significativamente menor que el del grupo de control^(19)^. Bayat et al.^(30)^ informaron que el entrenamiento cognitivo-conductual disminuyó la ansiedad de las mujeres embarazadas con resultados positivos en las pruebas de detección de trastornos cromosómicos. Çakar et al.^(25)^ observaron que las pacientes que obtenían información de médicos o enfermeras presentaban niveles de ansiedad significativamente más bajos. Cloutier et al.^(31)^ evaluaron los efectos del asesoramiento genético grupal en poblaciones de cribado prenatal de alto riesgo y encontraron que las mujeres que recibieron asesoramiento genético individual experimentaron una reducción significativa de la ansiedad en comparación con las sesiones grupales. Mojahed et al.^(32)^ analizaron el efecto de la educación sobre la ansiedad de las mujeres embarazadas antes de la amniocentesis y encontraron que los niveles de ansiedad del grupo de intervención que recibió la educación fueron significativamente más bajos que los del grupo de control. De manera similar, el ensayo controlado aleatorio realizado por Chuenwattana et al., que estudió el impacto de la información de apoyo en los niveles de ansiedad de las mujeres embarazadas que esperaban los resultados de la amniocentesis, reveló niveles de ansiedad significativamente más bajos en el grupo de intervención^(14)^. El coeficiente alfa de Cronbach para el STAI-S fue de 0,77 en el presente estudio. Aunque este valor es ligeramente inferior a los reportados en estudios de validación previos, se mantiene dentro del rango aceptable para fines de investigación e indica una consistencia interna satisfactoria para evaluar la ansiedad de estado en esta muestra. El estudio reveló que las mujeres embarazadas experimentan ansiedad debido a posibles intervenciones invasivas, preocupaciones sobre la salud y anomalías fetales, y el temor a la pérdida del feto. El asesoramiento genético prenatal puede reducir la ansiedad al brindar información personalizada, apoyo emocional y facilitar la toma de decisiones informadas durante el proceso de pruebas prenatales.

Aunque las puntuaciones iniciales del PSDTAQ difirieron significativamente entre los grupos de intervención y de control, tanto el modelo lineal de efectos mixtos como el ANCOVA demostraron que el efecto de la intervención se mantuvo estadísticamente significativo después de ajustar por la puntuación inicial, los ingresos, el nivel educativo y la edad gestacional. Estos hallazgos indican que la mejora observada en las actitudes hacia las pruebas de detección y diagnóstico prenatales fue sólida y no se debió a desequilibrios iniciales entre los grupos. Al confirmar la hipótesis H_1_, este estudio sugiere que el asesoramiento genético prenatal tiene un impacto positivo en las actitudes de las mujeres embarazadas hacia las pruebas de detección y diagnóstico prenatales. En consonancia con nuestros resultados, el único ensayo controlado aleatorio que utilizó el PSDTAQ para evaluar los efectos del asesoramiento genético prenatal en Turquía encontró un aumento significativo en las puntuaciones de las participantes en el PSDTAQ después del asesoramiento^(19)^. Por lo tanto, el asesoramiento genético prenatal puede mejorar las actitudes de las mujeres embarazadas hacia las pruebas de detección y diagnóstico prenatales al facilitar un asesoramiento informado y de apoyo durante el proceso de toma de decisiones.

## LIMITACIONES

Este estudio presenta varias limitaciones. En primer lugar, se llevó a cabo en un solo centro con un tamaño de muestra relativamente pequeño, lo que limita su generalizabilidad. En segundo lugar, no fue posible mantener el enmascaramiento de las participantes ni del proveedor de la intervención, lo que introduce la posibilidad de sesgos de atención y de expectativa. En tercer lugar, aunque las puntuaciones iniciales del PSDTAQ y el nivel de ingresos diferían entre los grupos, los análisis ajustados mediante ANCOVA y modelos lineales mixtos mostraron resultados consistentes. Por último, solo se evaluaron los resultados a corto plazo. Se justifica la realización de estudios futuros que empleen intervenciones de control con atención equiparada para distinguir el efecto específico del asesoramiento genético prenatal del efecto de una mayor interacción profesional.

## CONCLUSIONES

Los hallazgos de este estudio sugieren que se puede ofrecer asesoramiento genético prenatal durante las primeras etapas del embarazo para apoyar a las mujeres embarazadas que se someten a pruebas de detección y diagnóstico prenatales. El asesoramiento genético prenatal a cargo de enfermeras parece reducir la ansiedad y mejorar las actitudes hacia las pruebas de detección prenatal a corto plazo. Estos hallazgos deben confirmarse en ensayos aleatorios multicéntricos más amplios con un seguimiento más prolongado antes de formular recomendaciones educativas o de políticas de alcance general. Es imprescindible que las enfermeras comprendan el tipo, el propósito, la administración y la duración de las evaluaciones de diagnóstico prenatal, además de las posibles opciones a seguir tras conocer los resultados. En futuras investigaciones educativas se debe explorar la incorporación del asesoramiento genético prenatal en la formación de enfermería.

## Data Availability

El conjunto de datos que respaldan las conclusiones de este estudio está disponible previa solicitud al autor correspondiente.
